# PET/CT imaging of bone disorders in dysparathyroidism: a comprehensive review

**DOI:** 10.3389/fnume.2025.1645497

**Published:** 2025-10-23

**Authors:** E. Panagiotidis, G. Angelidis, V. Valotassiou, I. Tsougos, P. Georgoulias, J. T. Zhang-Yin

**Affiliations:** ^1^ Nuclear Medicine Laboratory, University of Thessaly, University Hospital of Larissa, Larissa, Greece; ^2^ Department of Nuclear Medicine, Clinique Sud Luxembourg, Arlon, Belgium; ^3^ Department of Nuclear Medicine, Centre National PET, Luxembourg, Luxembourg

**Keywords:** bone metabolism, brown tumors, fluorine-18 sodium fluoride ([18F]NaF), fluorine-18 fluorodeoxyglucose ([18F]FDG), fluorine-18 fluorocholine ([18F]FCH), gallium-68, prostate-specific membraneantigen ([68Ga]PSMA), dysparathyroidism

## Abstract

Parathyroid disorders profoundly affect bone metabolism, often long before structural damage is apparent on conventional imaging. Positron emission tomography/computed tomography (PET/CT) has emerged as a transformative tool in dysparathyroidism, enabling visualization of early metabolic bone changes and accurate localization of parathyroid pathology. This review explores the pathophysiology of bone disease in hyper- and hypoparathyroidism and highlights the role of key PET radiotracers: fluorine-18 sodium fluoride ([18F]NaF), fluorine-18 fluorodeoxyglucose ([18F]FDG), fluorine-18 fluorocholine ([18F]FCH), gallium-68 prostate-specific membrane antigen ([68Ga]PSMA). Distinct imaging patterns, such as the [18F]NaF “superscan” in secondary hyperparathyroidism and focal uptake in brown tumors, are discussed alongside tracer-specific strengths. Clinical applications including diagnosis, monitoring response to therapy, and prognostication are examined. We also consider emerging technologies such as artificial intelligence (AI)-assisted interpretation and positron emission tomography/magnetic resonance imaging (PET/MRI) fusion imaging. As PET/CT becomes more accessible, it is likely to play an increasingly central role in the early detection and personalized management of parathyroid-related bone disease.

## 1 Introduction

Parathyroid hormone (PTH) plays a central role in maintaining calcium and phosphate homeostasis, exerting direct effects on osteoblasts and indirect activation of osteoclasts via the receptor activator of nuclear factor kappa-B ligand (RANKL) pathway. Dysregulation of PTH—whether through excess secretion in hyperparathyroidism or deficiency in hypoparathyroidism—leads to distinct metabolic alterations that compromise skeletal integrity, even before visible structural damage appears (1–3).

Historically, our understanding of parathyroid-related bone disease dates to the late 19th century. Von Recklinghausen first described skeletal manifestations such as osteitis fibrosa cystica in 1891, though the hormonal origin of these changes was not understood until the 20th century. The advent of nuclear imaging in the 1980s, beginning with thallium-technetium subtraction scintigraphy and later technetium-99 m methoxyisobutylisonitrile (99mTc-MIBI) scans in the 1990s, significantly advanced preoperative localization of parathyroid lesions (1, 2). Despite these innovations, conventional modalities such as dual-energy x-ray absorptiometry (DXA), x-rays, computed tomography (CT), and magnetic resonance imaging (MRI) remain limited to detecting late-stage architectural changes.

By contrast, PET/CT offers a powerful molecular imaging platform capable of visualizing functional abnormalities before anatomical disruption occurs. Bone-specific PET tracers like [18F]NaF and fluorine-18 fluorocholine ([18F]FCH), along with newer agents such as gallium-68 prostate-specific membrane antigen ([68Ga]PSMA), allow for targeted assessment of both parathyroid tissue and skeletal metabolism. [18F]NaF is highly sensitive for detecting skeletal metabolic activity, particularly in conditions like renal osteodystrophy, where diffuse cortical uptake and “superscan” patterns (diffusely increased skeletal uptake with reduced soft-tissue/renal visualization) may appear. However, direct comparisons to ⁹⁹^m^Tc-labeled bisphosphonates remain limited (4, 5). [18F]FCH has demonstrated high diagnostic accuracy for parathyroid adenoma localization, with meta-analyses reporting sensitivities between 91% and 94% and high specificity, even in small or ectopic lesions (6).

This review examines the utility of PET/CT imaging in parathyroid disorders with skeletal involvement, structured across four main sections: the pathophysiology of bone changes in hyper- and hypoparathyroidism; tracer-specific imaging findings and mechanisms; clinical roles in diagnosis, monitoring, and risk stratification; and future developments, including artificial intelligence and PET/MRI integration. Emphasis is placed on radiotracer-specific patterns, such as the [18F]NaF “superscan” in secondary hyperparathyroidism or the incidental detection of brown tumors on [18F]FCH or [18F]FDG scans, which have historically been misdiagnosed as skeletal metastases (7).

## 2 Pathophysiology of bone disorders in dysparathyroidism

### 2.1 Hyperparathyroidism

Hyperparathyroidism, particularly the primary hyperparathyroidism (PHPT), is the most common cause of excessive PTH secretion. In about 80%–85% of cases, it is due to a single parathyroid adenoma, while multiglandular hyperplasia accounts for another 10%–15%, and parathyroid carcinoma remains rare (<1%) (1, 3). Elevated PTH levels chronically stimulate osteoclastic bone resorption via the RANKL-osteoprotegerin (OPG) signaling axis, tipping the remodeling balance in favor of bone breakdown (8). In parallel, chronic PTH exposure impairs osteoblast activity, altering collagen crosslinking and bone matrix quality—effects that aren't reflected on bone densitometry alone (2).

Clinically, early PHPT may be completely asymptomatic or present with subtle signs such as generalized fatigue and low bone density. However, bone involvement progresses with time. Cortical bone is especially vulnerable; changes are most pronounced in the distal radius, femoral neck, and skull (4). High-resolution CT studies show that cortical porosity increases significantly even in mild, asymptomatic cases, explaining why patients with PHPT have up to a 2–3-fold increased risk of fractures compared to age-matched controls despite normal in bone mineral density (BMD) on DXA scans (4).

A hallmark of severe, untreated PHPT is the development of brown tumors—focal osteolytic lesions composed of fibroblasts, hemosiderin, and multinucleated giant cells. These lesions appear in up to 10%–15% of patients in areas with limited access to biochemical screening but are rare (<2%) in countries with routine calcium testing (9). The mandible, ribs, pelvis, and long bones are common sites, and they often mimic malignancy radiologically, leading to diagnostic confusion, especially when [18F]FDG-avid (7, 9).

In secondary hyperparathyroidism (SHPT), typically seen in chronic kidney disease (CKD), the stimulus for excess PTH is sustained hypocalcemia due to impaired phosphate excretion and low vitamin D levels (10). This leads to diffuse parathyroid hyperplasia rather than focal adenomas. Over 90% of patients with end-stage kidney disease develop some form of SHPT over time (2). Bone changes in SHPT are variable and include both high-turnover lesions—similar to those in PHPT—and adynamic bone disease, especially with overtreatment using vitamin D analogs or calcimimetics (2, 4).

Tertiary hyperparathyroidism (THPT) emerges when long-standing SHPT becomes autonomous, often after renal transplant. Despite normalization of calcium levels, the hyperplastic parathyroid glands continue secreting PTH. In about 30% of renal transplant recipients with persistent hypercalcemia, this evolution to THPT is observed (10). Occasional overexpression of the cyclin D1 (CCND1), formerly proto-oncogene PRAD1, has been identified in parathyroid hyperplasia in uremic patients, suggesting a possible neoplastic component in long-standing secondary or tertiary hyperparathyroidism (11).

### 2.2 Hypoparathyroidism

In contrast, hypoparathyroidism is characterized by chronically low PTH levels, most often due to neck surgery (up to 75%–80% of cases), but also from autoimmune causes, genetic syndromes, or infiltrative diseases (12). Without PTH, calcium mobilization from bone is impaired, leading to hypocalcemia and hyperphosphatemia. However, the resulting suppression of bone remodeling paradoxically causes an increase in BMD—particularly in the lumbar spine and femoral neck (13).

This increased BMD can be misleading. Bone biopsy and histomorphometry reveal a state of low bone turnover, reduced remodeling surfaces, and hypermineralization, making the skeleton denser but more brittle (14). In one study, patients with hypoparathyroidism had 50%–70% lower bone remodeling rates than age-matched healthy individuals. Trabecular bone appears thickened but disorganized, and collagen abnormalities have also been observed. As a result, vertebral fractures are reported even in patients with above-average BMD, particularly with disease duration beyond 10 years (14).

Radiologically, hypoparathyroid bone may appear sclerotic, and patients may develop calcifications in soft tissues, basal ganglia, kidneys, or dental roots. PET tracers like [18F]NaF or [18F]FDG show low or absent uptake in most skeletal regions, reflecting suppressed bone turnover (5). With the advent of recombinant human PTH (1–84) therapy (brand name Natpara), studies now focus on identifying patients who may benefit most—particularly those with marked skeletal suppression and high fracture risk (15) Table 1.

**Table 1:** PET/CT Imaging Findings by Parathyroid Disorder Type.

Disorder	Bone findings	Parathyroid findings	Key tracers	Clinical use
PHPT	Cortical bone loss; brown tumors (lytic, [18F]FDG-avid; focal [18F]NaF uptake	Hyperfunctioning adenoma, best seen with [18F]FCH	[18F]NaF [18F]FCH [18F]FDG	Adenoma localization; distinguishing brown tumors from metastases
SHPT	Diffuse high-turnover bone disease; [18F]NaF “superscan”	Diffuse parathyroid hyperplasia	[18F]NaF	Skeletal disease assessment; metabolic activity monitoring
THPT	Persistent uptake post-transplant; adenoma/hyperplasia	Autonomous adenomas despite normocalcemia	[18F]NaF [18F]FCH,	Post-transplant evaluation; detection of autonomous lesions
Hypoparathyroidism	Osteosclerosis; soft tissue calcifications; low [18F]NaF, [18F]FDG uptake	Low/absent uptake in parathyroid tissue	[18F]NaF [18F]FDG	Identify suppressed bone turnover; therapy stratification (rhPTH candidates)

PHPT, primary hyperparathyroidism; SHPT, secondary hyperparathyroidism; THPT, tertiary hyperparathyroidism.

## 3 PET/CT radiotracers for bone and parathyroid imaging

The value of PET/CT in dysparathyroidism lies in its ability to simultaneously assess parathyroid tissue and bone metabolism using specialized radiotracers. The four most commonly used include [18F]NaF, [18F]FDG, [18F]FCH, and [68Ga]PSMA.

### 3.1 [18f]NaF

[18F]NaF PET/CT is highly sensitive for assessing skeletal turnover, binding to areas of active mineralization through exchange with hydroxyapatite crystals to form fluorapatite. Its favorable kinetics—rapid plasma clearance, low-protein binding, and nearly 100% first-pass extraction at bone surfaces—make it ideal for evaluating bone metabolism in endocrine and metabolic disorders (5).

**Figure 1:**
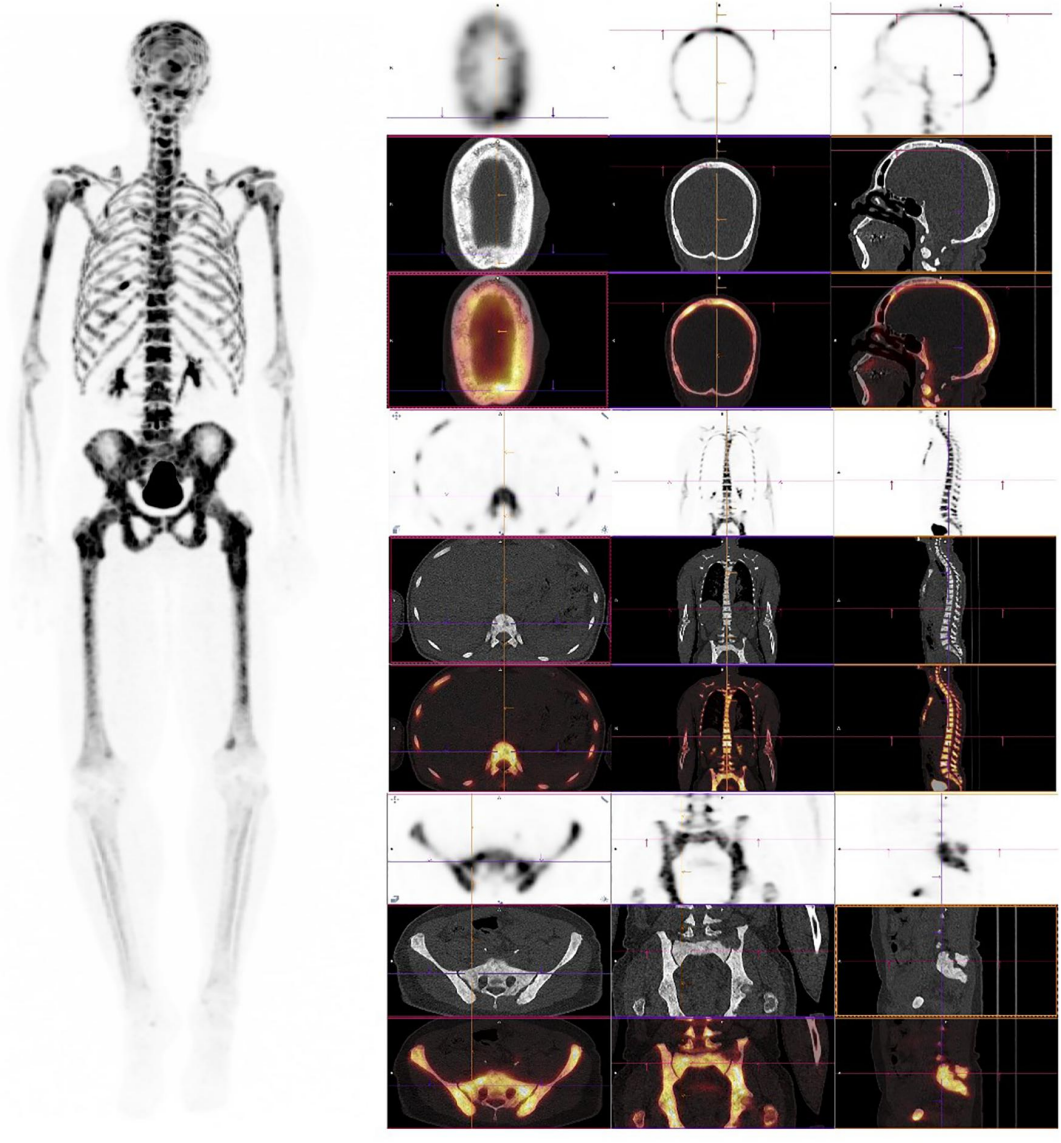
[18F]NaF PET/CT in secondary hyperparathyroidism with high-turnover metabolic bone disease. Left panel: Maximum intensity projection (MIP) shows diffusely increased skeletal uptake throughout the axial and appendicular skeleton with preserved renal visualization and prominent bladder activity, indicating normal tracer excretion rather than a classic superscan pattern. Right panels (low-dose CT and fused PET/CT): Cranial: Diffuse uptake in the calvarium and facial bones with a characteristic “salt-and-pepper” appearance on CT. Axial skeleton: Prominent uptake in vertebral bodies, ribs, and sternum, consistent with globally increased bone turnover. Appendicular skeleton: Increased uptake in the pelvis, femora, and other long bones, aligning with cortical-predominant involvement seen in renal osteodystrophy. The pattern reflects diffuse high-turnover bone disease associated with secondary hyperparathyroidism—generalized increased uptake on PET with diffuse sclerosis, skull “salt-and-pepper” changes, and cortical thickening on CT; preserved soft-tissue and renal visualization helps distinguish this from a true superscan presentation.

In parathyroid disorders, [18F]NaF reveals characteristic uptake patterns. In secondary and tertiary hyperparathyroidism, a “superscan” appearance is often observed, defined by diffusely increased skeletal uptake with diminished renal and soft tissue visualization. This pattern reported in up to 35% of patients with advanced renal osteodystrophy—reflects the global upregulation of bone turnover, particularly in cortical-rich sites such as the femoral shafts, pelvis, and ribs (16) Figure 1.

[18F]NaF is also susceptible to false-positive findings. In a comprehensive pictorial review, Panagiotidis et al. illustrated several benign skeletal conditions that may mimic metastatic disease, including fibrous dysplasia, healing fractures, Paget disease, and degenerative changes, highlighting the importance of integrating clinical, biochemical, and morphologic data to ensure accurate interpretation (17).

Quantitative [18F]NaF parameters (standardized uptake value (SUV), plasma clearance rate constant (K1) closely track with serum bone turnover markers such as alkaline phosphatase (ALP) and C-terminal telopeptide of type I collagen (CTX), with correlation coefficients ranging from *r* = 0.72–0.84 in prospective studies (18). Moreover, metabolic changes seen on [18F]NaF often precede changes in DXA by several months, offering an early window to evaluate treatment response after parathyroidectomy or PTH replacement (16).

Installe et al. evaluated the role of [18F]NaF in assessing treatment response in patients with Paget's disease of bone (19). In this prospective study, they reported a mean standardized uptake value (SUVmean) decrease of 65% in pagetic lesions after treatment, with baseline SUV values ranging from 12.3 to 51.6, and post-treatment values dropping to between 4.6 and 18.9. These metabolic changes strongly correlated with reductions in serum ALP (*p* < 0.001), demonstrating that [18F]NaF is a highly sensitive modality for quantitatively tracking therapeutic response in metabolic bone disease.

Conversely, in patients with suspected adynamic bone disease, [18F]NaF has demonstrated markedly reduced skeletal uptake. In a prospective study by Frost et al., mean “net influx rate constant (Ki)” values in hemodialysis patients were reduced by nearly 70% compared to healthy controls, confirming suppressed bone formation activity in renal osteodystrophy (20).

### 3.2 [18f]FDG

While [18F]FDG is primarily used for oncology, it also accumulates in inflammatory and reparative tissues—making it relevant in cases of brown tumors and active bone lesions. However, [18F]FDG uptake is nonspecific, and can be misleading, especially in oncologic patients where brown tumors mimic skeletal metastases. [18F]FDG-avid brown tumors have been reported with SUVmax ranging from 4.5 to 12.3 (9, 21). In the case report by Tsushima et al., a 38-year-old man with parathyroid carcinoma presented with multiple osteolytic lesions on [18F]FDG, which were initially suspected to be skeletal metastases. However, a biopsy of a right scapular lesion confirmed it to be a brown tumor. This case underscores the importance of correlating PET findings with biochemical parameters, such as elevated PTH and calcium levels, to avoid misdiagnosis and unnecessary interventions (22). In such contexts, correlating findings with [18F]NaF or biochemical parameters (elevated PTH, calcium) is critical to avoid unnecessary biopsy or treatment delays (23).

Interestingly, parathyroid adenomas show variable [18F]FDG avidity. A small percentage are intensely [18F]FDG-avid which may indicate higher cellular proliferation or, in rare cases, parathyroid carcinoma (24). Glucose transporter 1 (GLUT-1) and hexokinase II overexpression in such lesions has been confirmed in histopathologic studies, explaining this behavior (25).

In a case of osteitis fibrosa cystica generalisata due to untreated primary hyperparathyroidism, Holzgreve et al. demonstrated intense [18F]FDG uptake in multiple skeletal lesions on PET/CT, mimicking widespread metastases, while 99mTc-MIBI single-photon emission computed tomography/computed tomography (SPECT/CT) identified the underlying parathyroid adenoma (26). In contrast, Tsai et al. reported a patient with benign parathyroid hyperplasia and pulmonary parathyroid tissue seeding showing 99mTc-MIBI uptake but no [18F]FDG avidity, thus avoiding misdiagnosis of metastatic spread (27). These cases highlight the variable [18F]FDG avidity of parathyroid-related lesions and underscore the importance of correlating PET/CT findings with biochemical markers and clinical history to distinguish between benign hyperfunctioning tissue and malignancy.

In a 2024 case report by Mohd Rohani et al., a patient with secondary hyperparathyroidism exhibited a rare metabolic superscan pattern on [18F]FDG, characterized by diffusely increased skeletal uptake and suppressed soft tissue background. This finding, although typically associated with ⁹⁹^m^Tc bone scintigraphy, highlights how severe, high-turnover bone disease in renal osteodystrophy can occasionally manifest as a superscan on [18F]FDG imaging as well (28).

### 3.3 [18f]FCH

[18F]FCH is currently one of the most accurate imaging tools for localizing hyperfunctioning parathyroid adenomas. The mechanism is based on increased phospholipid synthesis in metabolically active parathyroid cells via upregulated choline kinase.

A high-quality meta-analysis by Whitman et al. compared [18F]FCH with 99mTc-MIBI for the preoperative localization of parathyroid adenomas (29). Their pooled analysis of 23 studies found that [18F]FCH demonstrated superior patient-level sensitivity (92%; 95% CI: 88%–95%) compared to 99mTc-MIBI (59%; 95% CI: 46%–71%). However, the authors noted considerable heterogeneity among studies, and emphasized that differences in imaging protocols, reference standards, and patient selection criteria may influence these results.

A systematic review by Broos et al. assessed the diagnostic performance of [18F]FCH for parathyroid adenoma localization in primary hyperparathyroidism (30). The pooled detection rate across 11 studies was 97% per patient and 94% per lesion, suggesting high diagnostic accuracy. However, the authors noted considerable heterogeneity and rated the level of evidence as 3a- according to Oxford criteria, indicating that results should be interpreted with caution.

A 2023 meta-analysis by Quak et al. evaluated the performance of [18F]FCH in primary hyperparathyroidism, reporting a patient-based sensitivity of 93.8% (95% CI: 89.8–96.3), positive predictive value (PPV) of 97% (95% CI: 92.8–98.8), and a cure rate of 92.8% (95% CI: 87.4–96.0) following PET-guided surgery (6). These results highlight the high diagnostic accuracy of [18F]FCH for preoperative localization of hyperfunctioning parathyroid glands and its potential to improve surgical outcomes.

A 2024 randomized clinical trial by Quak et al. compared [18F]FCH with 99mTc-MIBI in patients undergoing surgery for primary hyperparathyroidism (31). Among 132 patients, [18F]FCH achieved significantly higher lesion localization rates (94.1% vs. 57.6%, *P* < .001), leading to more frequent minimally invasive parathyroidectomy. The authors concluded that [18F]FCH offers superior preoperative guidance compared to conventional imaging. These findings strengthen the clinical utility of [18F]FCH as a first-line imaging modality in PHPT, particularly when surgery is planned.

A 2025 prospective study by Garnier et al. evaluated [18F]FCH PET/CT in 47 patients undergoing surgery for primary hyperparathyroidism (32). The modality successfully localized parathyroid adenomas in 93.6% of cases, showing strong concordance with surgical findings and histopathology. In 82.9% of patients, the PET/CT findings directly guided focused, minimally invasive parathyroidectomy. These results confirm the added clinical value of [18F]FCH in routine endocrine practice, especially where high diagnostic precision is required.

Pasini Nemir et al. investigated the diagnostic utility of [18F]FCH in 31 patients with biochemically confirmed PHPT who had negative or inconclusive cervical ultrasound and 99mTc-MIBI (33). [18F]FCH correctly localized the pathological gland in 90.3% of cases, enabling curative surgery in the majority of patients. The study reinforces the role of [18F]FCH as a reliable second-line imaging tool in challenging or discordant diagnostic settings.

A prospective dual-centre study by Beheshti et al. assessed the diagnostic performance of [18F]FCH vs. 99mTc-MIBI or 99mTc-tetrofosmin SPECT/CT in 100 patients with biochemically confirmed primary hyperparathyroidism (34). On a per-patient basis, [18F]FCH achieved a sensitivity of 92.3%, compared to 67.3% for SPECT/CT, with lesion-based sensitivity also significantly higher for [18F]FCH (91.6% vs. 63.2%). The authors concluded that [18F]FCH not only improves localization accuracy but may also increase the likelihood of successful minimally invasive surgery, especially in patients with negative or inconclusive conventional imaging.

In a prospective study of 44 patients with primary hyperparathyroidism and inconclusive first-line imaging, Piccardo et al. demonstrated that integrated [18F]FCH four-dimensional contrast-enhanced computed tomography (PET/4D-CeCT) achieved a detection rate of 72.7% and a sensitivity of 100% in surgically confirmed cases (35). These results were significantly superior to [18F]FCH alone (56.8% detection, 80% sensitivity) and to 4D-CeCT alone (54.5% detection, 74% sensitivity). The study also found that choline uptake correlated with calcium levels and Ki-67 expression, suggesting a link between imaging findings and molecular tumor characteristics.

Boccalatte et al. conducted a systematic review to assess the diagnostic accuracy of [18F]FCH for localizing hyperfunctioning parathyroid tissue in patients with hyperparathyroidism (36). Across 17 included studies comprising 745 patients, the pooled sensitivity of [18F]FCH was 92.6%**,** and specificity was 93.2%**,** with the modality outperforming conventional imaging in most comparative studies. The authors concluded that [18F]FCH is a highly effective second-line imaging tool, especially useful in cases with inconclusive ultrasound or 99mTc-MIBI scans.

**Figure 2:**
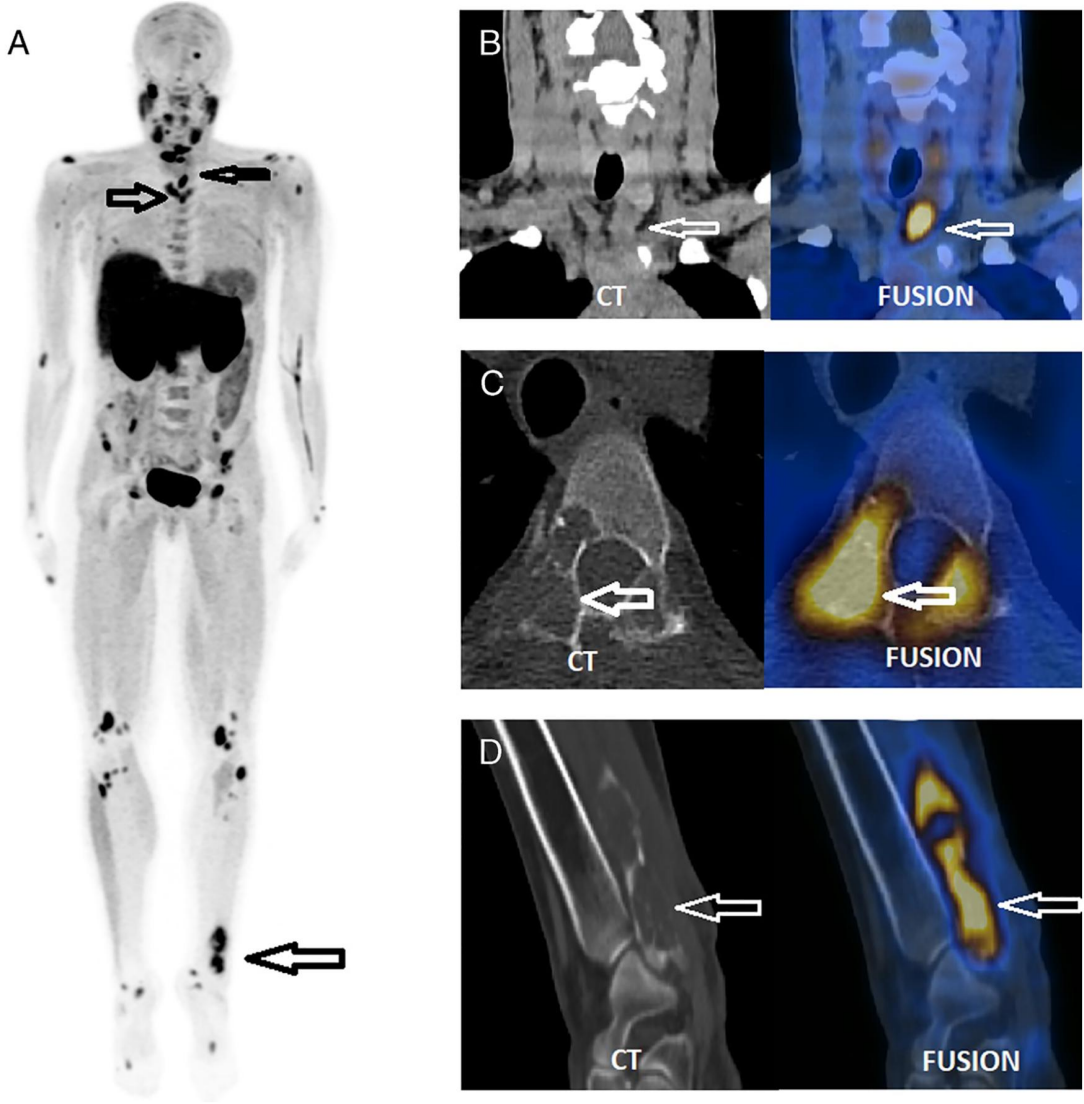
A single focus of increased [18F]FCH uptake was observed inferior to the left thyroid lobe (SUVmax 8.3; CT long axis: 28 mm), suggestive of a hyperfunctioning parathyroid adenoma **(B)** Multiple osteolytic lesions were identified on CT in the cervical region within the PET/CT field of view **(C)** The maximum intensity projection (MIP) image from the [18F]FCH PET/CT scan **(A)** revealed additional abnormal skeletal foci, confirmed as osteolytic on CT **(C,D)**, and more extensive than those visualized on prior bone scintigraphy. Surgical excision confirmed a left inferior parathyroid adenoma. Histological analysis of a biopsy from the painful left distal fibular lesion revealed a brown tumor, corresponding to the [18F]FCH-avid osteolytic site seen on PET/CT **(D)**. Reproduced with permission from “FCH PET/CT, which benefits from the superior resolution of PET compared with SPECT, was performed to guide parathyroidectomy” by Jules Zhang-Yin, Sébastien Gaujoux, Thierry Delbot, Mathieu Gauthé, Jean-Noël Talbot, licensed under CC BY-NC-ND.

Beyond its well-established role in localizing parathyroid adenomas, [18F]FCH has also shown promise in detecting skeletal manifestations of hyperparathyroidism, particularly brown tumors. Zhang-Yin et al. (2019) reported the first documented case in which [18F]FCH concurrently identified a hyperfunctioning parathyroid adenoma and multiple osseous lesions in a young patient with primary hyperparathyroidism (Figure 2) (37). These bone lesions, subsequently confirmed as brown tumors on histological analysis, demonstrated avid [18F]FCH uptake, underscoring the tracer's affinity for metabolically active bone. The imaging provided a comprehensive, “one-stop” evaluation, facilitating both preoperative planning and lesion characterization. Follow-up imaging after parathyroidectomy revealed partial regression of the osseous lesions, reinforcing their metabolic etiology, and highlighting the clinical value of [18F]FCH in the early and accurate assessment of skeletal involvement in hyperparathyroidism.

### 3.4 [68Ga]PSMA

[68Ga]PSMA, widely used in prostate cancer imaging, has shown promise in localizing parathyroid adenomas—particularly in cases with equivocal or failed conventional imaging. PSMA expression has been detected not only in prostate tissue but also in the neovasculature of hyperfunctioning parathyroid glands. [68Ga]PSMA PET/CT has occasionally revealed incidental uptake in parathyroid adenomas, although evidence remains limited to case reports and small series, and its role is therefore investigational rather than established.

Cieślewicz et al. reported a case of primary hyperparathyroidism in which the parathyroid adenoma was not visualized by conventional imaging modalities including ultrasound, 99mTc-MIBI, and [18F]FCH (38). Remarkably, the lesion was successfully localized using [68Ga]PSMA, likely due to PSMA expression in the tumor's neovasculature. This case highlights the potential utility of PSMA-targeted imaging in challenging parathyroid cases, particularly when standard methods fail.

Another case report by Pfob et al. described the incidental detection of a parathyroid adenoma on a [68Ga]PSMA scan performed for prostate cancer staging. The lesion, located inferior to the thyroid gland, exhibited intense PSMA uptake and was later confirmed histologically as a parathyroid adenoma, highlighting a potential pitfall in PSMA imaging, as benign parathyroid lesions may mimic metastatic disease due to unexpected tracer avidity (39). Broos et al. described an incidental parathyroid adenoma detected on [18F]PSMA during prostate cancer imaging. The lesion showed intense PSMA uptake and was later confirmed as a benign parathyroid adenoma, illustrating a potential diagnostic pitfall (40).

Although PSMA uptake in bone lesions (e.g., brown tumors) is less well-characterized, the dual ability of this tracer to assess both parathyroid and osseous involvement represents a compelling area for future research. However, the uptake of PSMA in brown tumors remains poorly understood, with limited literature exploring this phenomenon (21).

### 3.5 Emerging PET tracers and applications

Historically, other tracers beyond the main agents discussed have been explored for parathyroid imaging. For instance, the utility of [18F]DOPA was assessed in a pilot study by Lange-Nolde et al. in 2006 (41). In that small cohort, it was suggested that [18F]DOPA could localize parathyroid adenomas when conventional imaging was inconclusive. However, this tracer has not been widely adopted for this indication due to limited subsequent evidence and the superior, well-documented performance of agents such as [18F]FCH.

Evangelista et al. conducted a systematic review evaluating the diagnostic performance of [18F]FCH PET/CT and PET/MRI in both primary and recurrent hyperparathyroidism (42). Across 23 studies, they reported pooled detection rates ranging from 89% to 97% for [18F]FCH and highlighted its superior sensitivity compared to conventional modalities, particularly in reoperative cases. While the review highlighted the potential of PET/MRI, further data are needed to confirm its clinical value.

It should be noted that while Carbon-11 labeled tracers, such as [^11^C]methionine and [^11^C]choline, have also been investigated for parathyroid imaging, their clinical application is significantly limited. The very short half-life of Carbon-11 (∼20 min) necessitates an on-site cyclotron for production, which restricts their availability to highly specialized centres. Consequently, Fluorine-18 based agents, with their longer half-life and wider availability, are generally favored in clinical practice.

Kuyumcu et al. evaluated [68Ga]Trivehexin, a novel PET tracer targeting integrin alpha-v beta-6 (αvβ6), for the detection of parathyroid adenomas in patients with primary hyperparathyroidism (43). In this prospective study, [68Ga]Trivehexin PET/CT successfully identified parathyroid lesions in over 90% of patients, including cases with negative conventional imaging. These findings suggest that αvβ6-targeted imaging may represent a new molecular approach for accurate localization in challenging parathyroid cases.

A novel bisphosphonate-based tracer, [68Ga]P15-041, has shown excellent performance in detecting skeletal metastases, with a reported sensitivity of 93.1% and accuracy of 90.7%—exceeding that of conventional bone scintigraphy and offering high-contrast, rapid imaging as a potential alternative to [18F]NaF in resource-limited settings (44).

Recent studies have highlighted the utility of [18F]aluminum fluoride NOTA-octreotide (18F-AlF-NOTA-octreotide) in localizing culprit tumors in tumor-induced osteomalacia (TIO), a condition that often poses significant diagnostic challenges. This somatostatin receptor-targeted tracer provides high lesion-to-background contrast, facilitating the detection of small mesenchymal tumors even in anatomically complex regions. A 2021 case series and literature review by Long et al. demonstrated diagnostic performance comparable to that of [68Ga]DOTA-Tyr3-octreotate (68Ga-DOTATATE), while offering logistical advantages in centers lacking access to a gallium generator. These findings support the adoption of 18F-AlF-NOTA-octreotide as a promising alternative in the evolving molecular imaging armamentarium for metabolic bone disorders (45). Ongoing research also explores tracers targeting osteoclasts—such as radiolabeled cathepsin K inhibitors—and matrix remodeling proteins, which may enable PET imaging to advance beyond metabolic turnover toward microstructural and qualitative assessment of bone pathology (46) Table 2.

**Table 2:** Advantages of different PET tracers in dysparathyroidism.

Tracer	Primary application	Secondary application
[18F]NaF	Bone turnover assessment	Treatment monitoring
[18F]FDG	Brown tumor identification	Parathyroid carcinoma evaluation
[18F]FCH	Parathyroid adenoma localization of bone lesions	Incidental detection
[68Ga]PSMA	Challenging adenoma localization applications; potential use in atypical adenomas	Research

## 4 PET/CT findings in dysparathyroidism: pattern recognition and clinical correlation

PET/CT imaging patterns in dysparathyroidism vary significantly based on the underlying hormonal dysfunction—whether driven by excess or deficiency of parathyroid hormone. Recognition of tracer-specific uptake patterns not only supports diagnosis but can also influence management decisions.

### 4.1 Primary hyperparathyroidism (PHPT)

Brown tumors may appear as focal lesions with intense [18F]NaF uptake and corresponding lytic changes on CT. In one series, SUVmax values in brown tumors ranged up to an SUVmax of 12.3, and histologic confirmation revealed giant-cell-rich stroma consistent with the diagnosis (21). Importantly, [18F]NaF helps distinguish metabolically active bone lesions from chronic or healed abnormalities, particularly in patients with multiple skeletal findings.

In the context of parathyroid adenoma localization, [18F]FCH has demonstrated superior diagnostic accuracy, especially in patients with inconclusive conventional imaging. In approximately 20%–25% of patients with negative or discordant findings on ultrasound and 99mTc-MIBI, [18F]FCH was able to successfully localize hyperfunctioning parathyroid tissue, leading to changes in surgical planning and approach (33, 34).

### 4.2 Secondary and tertiary hyperparathyroidism (SHPT/THPT)

In SHPT, which frequently occurs in chronic kidney disease, [18F]NaF often reveals a “superscan” appearance—markedly diffuse skeletal uptake with minimal background activity, however, current evidence derives mainly from small case series, and prospective multicenter validation is still lacking. This reflects global high-turnover bone disease, which remains prevalent in patients on dialysis despite therapy. One prospective PET study in dialysis patients showed SUVmean values increased by 35%–50% compared to healthy controls, and uptake was significantly correlated with serum ALP (*r* = 0.78) (20).

Tertiary hyperparathyroidism, most often seen post-renal transplant, can produce similar imaging findings. [18F]NaF uptake often persists even after parathyroidectomy, likely due to slow remodeling of the sclerotic renal bone. In a longitudinal study of post-transplant patients, skeletal [18F]NaF uptake remained elevated for over 12 months in 42% of patients despite biochemical resolution of PTH excess (16). This ongoing skeletal remodeling imbalance may be further mediated by wingless/integrated (Wnt) pathway inhibitors such as sclerostin and Dickkopf-1 (DKK1), which are upregulated in CKD and contribute to both bone fragility and vascular calcification (47).

[18F]FCH is useful in tertiary cases with persistent hypercalcemia to detect autonomous adenomas. Studies show a sensitivity of approximately 85% in this context, especially when prior imaging was negative (31, 33).

### 4.3 Hypoparathyroidism

In hypoparathyroidism, bone turnover is significantly reduced due to the absence of PTH. This is reflected on [18F]NaF by low skeletal uptake, particularly in trabecular regions such as the vertebrae and pelvis. In a cohort of 21 patients with post-surgical hypoparathyroidism, SUVmean in the lumbar spine was nearly 40% lower than in age- and sex-matched controls (mean 4.2 vs. 6.8; *p* < 0.01), consistent with suppressed remodeling (20).

Interestingly, despite higher BMD on DXA, several studies have reported increased vertebral fracture risk. One study involving 120 patients with long-standing hypoparathyroidism found that 19% had vertebral fractures, despite BMD T-scores >+1.0 in most cases (14). This disconnect between mineral content and biomechanical strength may be partially explained by abnormal microarchitecture—evident on bone biopsy and high-resolution imaging as hypermineralized, poorly connected trabeculae.

[18F]NaF offers valuable insight into this paradox. Low tracer uptake despite high BMD suggests skeletal quiescence and brittle bone quality, helping to identify patients who may benefit from recombinant human parathyroid hormone (1-84) [rhPTH(1-84)] therapy (15). Importantly, recent work by Siddique et al. has shown that static [18F]NaF images can reliably estimate regional bone metabolism, allowing routine, non-invasive assessment of skeletal turnover without dynamic imaging or arterial sampling (48). This makes [18F]NaF a practical tool in longitudinal monitoring of bone health in hypoparathyroid patients (20).

## 5 Clinical applications of PET/CT in dysparathyroidism

### 5.1 Diagnosis and localization: beyond conventional imaging

A critical clinical challenge in primary hyperparathyroidism is accurately locating the hyperfunctioning parathyroid gland(s), particularly in patients with small, ectopic, or multiglandular disease. While ultrasound and 99mTc-MIBI remain first-line tools, their sensitivity varies widely depending on operator skill and patient anatomy. Accurate localization not only improves surgical outcomes but also reduces the need for bilateral neck exploration, potentially lowering healthcare costs and complication rates (49, 50).

[18F]FCH offers substantial improvement in diagnostic performance over conventional modalities. A 2019 systematic review by Broos et al. found detection rates of 97% per patient and 94% per lesion, further underscoring the high accuracy of [18F]FCH in localizing hyperfunctioning parathyroid tissue, particularly in cases where conventional imaging is inconclusive or discordant (30). Moreover, a prospective study by Lezaic et al. demonstrated that the use of [18F]FCH enabled focused parathyroidectomy in 75% of patients, significantly reducing the need for bilateral neck exploration and thereby optimizing surgical planning and outcomes (51).

### 5.2 Evaluation of skeletal disease

Bone involvement in parathyroid disorders, particularly in asymptomatic patients, is often underrecognized. [18F]NaF has emerged as a sensitive modality for assessing regional bone turnover, offering higher spatial resolution and improved image contrast compared to traditional bone scintigraphy (52). This imaging technique allows for the detection of metabolic changes in bone before structural alterations become apparent on conventional imaging modalities.

In cases of suspected brown tumors or unexplained lytic lesions, PET/CT can help localize metabolically active sites. While increased uptake in these lesions is not specific for malignancy, it may prompt further evaluation, especially when correlated with elevated parathyroid hormone (PTH) levels and bone turnover markers. However, the role of PET/CT in guiding biopsies for such lesions remains to be fully established.

### 5.3 Monitoring treatment response

PET/CT imaging, particularly with [18F]NaF, shows promise in monitoring the skeletal response to therapy in parathyroid disorders. Serial [18F]NaF scans can detect changes in bone metabolism following parathyroidectomy, often before improvements are observed in BMD measurements. A study demonstrated that tracer uptake measured in bone grafts decreased over time, with 25% decrease six months after surgery and a 60%–65% decrease two years after surgery (53).

In patients with CKD, [18F]NaF has been used to assess bone turnover, showing significant differences in uptake between patients with low and high bone turnover states. This suggests potential utility in evaluating treatment response in secondary hyperparathyroidism, although further studies are needed to validate these findings (54).

### 5.4 Prognostication and risk stratification

PET/CT may also offer prognostic insights. Quantitative analysis of [18F]NaF uptake has shown promise for assessing bone turnover and skeletal integrity beyond what is measurable by BMD alone. In a 2024 systematic review, de Ruiter et al. confirmed that SUV-based metrics such as SUVmean and SUVmax correlate well with full kinetic modeling across various bone diseases, supporting their use as non-invasive surrogates of metabolic activity (55). Although further clinical studies are needed, such quantification may eventually aid in stratifying fracture risk and identifying patients who require closer follow-up or earlier therapeutic intervention.

Rhodes et al. evaluated the use of [18F]NaF imaging to quantify bone metabolism at the femoral neck through a novel metric called the Bone Metabolism Score (BMS) (56). This parameter integrates SUV measurements and anatomical localization to reflect regional skeletal activity. The study demonstrated strong correlations between BMS, age, and BMD, highlighting its relevance as a surrogate marker of bone health. These findings suggest that [18F]NaF may help identify individuals at higher risk of fragility fractures, even before structural deterioration becomes evident on DXA (Table 3).

**Table 3:** Clinical applications of PET/CT in dysparathyroidism.

Application	PET/CT contribution
Localization of parathyroid adenomas	High-resolution detection with [18F]FCH or [68Ga]PSMA PET/CT
Evaluation of bone disease	Differentiates brown tumors, osteitis fibrosa cystica, sclerosis
Therapy monitoring	Tracks metabolic response post-parathyroidectomy
Prognostication	Uptake correlates with disease activity and fracture risk
Incidental findings	Detects subclinical skeletal involvement

## 6 Advantages and limitations of PET/CT

The integration of PET/CT into the evaluation of parathyroid-related bone disease has changed how clinicians approach diagnosis and management. Its unique ability to image function rather than just structure gives it a decisive advantage over conventional imaging tools like CT, MRI, or bone scintigraphy.

A major benefit of PET/CT is its capacity for early disease detection. Changes in bone metabolism can be identified months before structural abnormalities appear on standard imaging. For example, [18F]NaF has been shown to reveal increased bone turnover as early as 6–8 months prior to changes on radiographs or DXA scans, offering an opportunity for earlier intervention and potentially preventing irreversible damage (53).

Diagnostic accuracy is another key strength. In localizing parathyroid adenomas, [18F]FCH consistently outperforms other modalities. Large meta-analyses report a pooled sensitivity above 90%, far higher than 99mTc-MIBI (around 69%) or ultrasound (around 76%). This translates into more accurate surgical planning, reduced operative time, and fewer complications. In some centers, the use of [18F]FCH has reduced the need for bilateral neck exploration by nearly half (51).

For bone disease assessment, [18F]NaF offers a quantitative view of skeletal metabolism. Uptake values such as SUVmax or SUVmean reflect osteoblastic activity and can be correlated with serum markers like alkaline phosphatase or PTH. In patients with secondary hyperparathyroidism, studies have shown a strong correlation (*r* = 0.78) between [18F]NaF uptake and ALP levels, allowing clinicians to evaluate disease severity and monitor response to treatment (53). Notably, metabolic improvement on PET/CT often precedes measurable changes in bone density, helping clinicians assess the effectiveness of therapy earlier (53).

Despite these advantages, PET/CT does have limitations. Access to advanced radiotracers like [18F]FCH or [68Ga]PSMA remains uneven, often restricted to academic or research centers. Cost is also a consideration, as PET/CT is more expensive than traditional nuclear medicine scans. However, there is growing evidence that its higher diagnostic yield may ultimately reduce total costs by improving surgical precision and reducing unnecessary procedures (49). The widespread use of PET/CT in dysparathyroidism remains constrained by tracer availability, cost-effectiveness considerations, and the absence of standardized acquisition and interpretation protocols. These factors currently restrict routine adoption outside specialized centers.

Radiation exposure, while generally within acceptable limits, is another factor—especially in younger patients or those requiring multiple scans. A typical [18F]NaF study delivers about 4–7 mSv, similar to two low-dose chest CTs (36). Risk-benefit analysis is essential in each case.

Interpretation of PET/CT results requires experience, particularly because benign lesions can mimic more serious conditions. For instance, degenerative changes, fibrous dysplasia, or healing fractures can appear suspicious on [18F]NaF scans. A 2018 pictorial review by Panagiotidis et al. emphasized this issue, presenting several benign conditions that may be mistaken for metastases if imaging is read in isolation (17). Clinical and biochemical context remains essential.

An important limitation of the current evidence base is its imbalance: [18F]FCH is supported by robust meta-analyses and large prospective studies, whereas [18F]NaF and [68Ga]PSMA data rely mainly on case reports and small pilot studies. This highlights both the promise and the need for systematic trials in these areas.

Finally, it's important to recognize that some of the most promising applications of PET/CT in parathyroid disease—such as PSMA or DOPA imaging—are still considered investigational in many settings. While research supports their use, official guidelines and insurance coverage may not yet reflect this, posing regulatory and logistical hurdles for broader clinical adoption.

## 7 Future perspectives

The role of PET/CT in the management of dysparathyroidism is still evolving. While it has already improved our ability to detect disease early and guide treatment decisions, ongoing innovations in molecular imaging, artificial intelligence (AI), and hybrid technologies are expected to expand its impact even further.

One of the most promising areas of development involves the next generation of radiotracers. Beyond the widely used [18F]NaF and [18F]FCH, researchers are investigating tracers that can target specific aspects of bone biology. For example, a novel bisphosphonate-based tracer, [68Ga]P15-041, has shown excellent performance in detecting skeletal metastases, with a reported sensitivity of 93.1% and accuracy of 90.7%—exceeding that of conventional bone scintigraphy and offering high-contrast, rapid imaging as a potential alternative to [18F]NaF in resource-limited settings (44). Similarly, radiotracers targeting osteoclast activity—such as cathepsin K inhibitors—could eventually enable direct visualization of bone resorption, something current tracers cannot do. Additionally, [18F]DOPA, best known for neuroendocrine tumor imaging, has shown early promise in parathyroid adenoma localization, with reported sensitivities approaching 90% in small clinical studies (41).

Another frontier lies in the integration of AI. Machine learning algorithms are being trained to detect subtle abnormalities, differentiate physiological from pathological uptake, and even predict treatment response based on image patterns. In early studies, AI-assisted PET/CT interpretation has demonstrated higher sensitivity for detecting small or ectopic parathyroid adenomas, particularly when combined with clinical and biochemical data (53). AI may also help standardize image quantification across centers and automate lesion segmentation, streamlining workflows in busy clinical settings (57).

Radiomics—the extraction of quantitative imaging features not visible to the human eye—is another exciting approach. Applied to PET/CT, it could allow prediction of fracture risk, assessment of bone microstructure, or even classification of lesion types based on uptake texture and shape. Though still in early stages, radiomics applications in bone PET imaging are being actively studied and could soon become part of precision imaging pipelines (58, 59).

Hybrid imaging with PET/MRI is also attracting interest. While PET/CT remains the workhorse, PET/MRI offers superior soft tissue contrast and better delineation of structures like bone marrow, parathyroid glands, and ectopic calcifications. In patients with chronic dysparathyroidism, PET/MRI may improve characterization of brown tumors, marrow fibrosis, or calcifications in soft tissues and basal ganglia—especially when combined with advanced MRI sequences like diffusion-weighted imaging and dynamic contrast enhancement. Early reports suggest that PET/MRI could be particularly useful in children, young adults, or patients requiring repeated imaging, where radiation minimization is crucial (42).

Recent developments in digital PET/CT scanners have brought meaningful improvements in spatial resolution, contrast recovery, and time-of-flight (TOF) capabilities. These technical advances offer benefits for parathyroid and skeletal imaging applications. Digital PET technology enhances lesion detection, especially for small or ectopic parathyroid adenomas that conventional analog systems frequently fail to visualize. A 2024 review highlighted the improved signal-to-noise ratio and lesion-to-background contrast seen with digital systems compared to analog predecessors, supporting their expanding role in precise endocrine imaging (60).

These technological advantages prove especially valuable in challenging clinical scenarios such as multiglandular disease or recurrent hyperparathyroidism, where traditional imaging methods often produce inconclusive results. Moreover, digital PET/CT allows for faster scanning sequences and requires lower tracer doses, potentially improving patient experience while enabling broader application in radiation-sensitive populations, including pediatric and post-transplant patients. These advances represent a significant step forward in functional and anatomical imaging capabilities for endocrine disorders, particularly for difficult parathyroid conditions.

Despite the promising results of PET/CT tracers in dysparathyroidism, several limitations must be acknowledged. The widespread use of these modalities is constrained by tracer availability, cost-effectiveness considerations, and the absence of standardized acquisition and interpretation protocols. These factors currently restrict routine adoption outside specialized referral centers.

Guideline positioning reflects regional variability. The European Association of Nuclear Medicine (EANM) guidelines have acknowledged the emerging role of PET tracers, particularly [18F]FCH, in challenging cases of parathyroid imaging (61). In contrast, American recommendations, such as the American College of Radiology (ACR), and the National Comprehensive Cancer Network (NCCN) guidelines, continue to emphasize ultrasound and 99mTc-MIBI as standard approaches without integration of PET tracers (62). These differences highlight both regional variability in clinical adoption and the need for harmonization of practice as the evidence base for PET/CT in dysparathyroidism continues to expand.

## 8 Conclusion

PET/CT imaging, particularly with [18F]FCH, has emerged as a highly accurate tool for parathyroid adenoma localization and for assessing skeletal involvement in dysparathyroidism. Its diagnostic performance often surpasses conventional modalities, offering clear benefits in surgical planning and disease monitoring. However, current barriers—including limited tracer availability, cost-effectiveness concerns, and the lack of standardized imaging protocols—restrict its widespread adoption. Moreover, international guidelines remain inconsistent, with European recommendations beginning to recognize the role of PET tracers while American guidelines continue to emphasize traditional approaches.

Moving forward, harmonization of practice guidelines and prospective multicenter studies will be critical to define the place of PET/CT in the diagnostic and management algorithms of dysparathyroidism. Until then, PET/CT should be viewed as a powerful but still selectively applied tool, best utilized in complex cases where conventional imaging is inconclusive.
